# Enhancement of Mechanical Properties of High-Thermal-Conductivity Composites Comprising Boron Nitride and Poly(methyl methacrylate) Resin through Material Design Utilizing Hansen Solubility Parameters

**DOI:** 10.1021/acsami.4c00626

**Published:** 2024-05-09

**Authors:** Yumi Inagaki, Masakazu Murase, Hiromitsu Tanaka, Daisuke Nakamura

**Affiliations:** Toyota Central R&D Laboratories, Inc., Nagakute, Aichi 480-1192, Japan

**Keywords:** boron nitride, poly(methyl methacrylate), composite, Hansen solubility parameters, material characterization

## Abstract

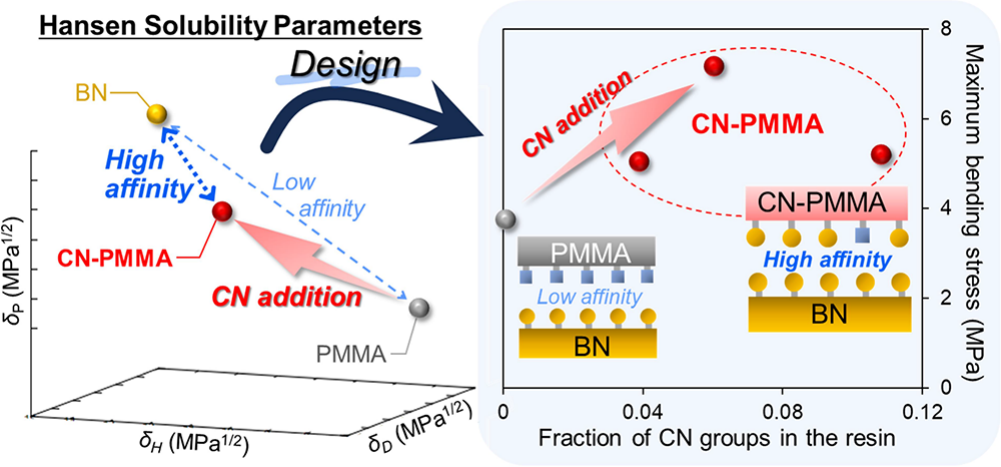

Materials for heat sinks in automotive heat dissipation systems must demonstrate both high thermal conductivity and stress resistance during assembly. This research proposes a composite material, comprised of thermally conductive ceramic fillers and matrix resins, as a suitable option for such application. The strategy for designing this material interface is directed with Hansen solubility parameters (HSP). A composite material featuring a honeycomb-like structure made of poly(methyl methacrylate) (PMMA) and boron nitride (BN) particles was successfully fabricated through press molding. This yielded a continuous BN network exhibiting high thermal conductivity and moderate mechanical strength. The HSP evaluation led to the suggestion of introducing highly polar functional groups into the matrix resin to enhance the affinity between PMMA resin and BN fillers. In line with this recommendation, a nitrile (CN) group—a highly polar group—was introduced to PMMA (CN-PMMA), significantly enhancing the composite’s maximum bending stress without noticeably degrading other properties. Surface HSP evaluation through contact angle measurements revealed an “interface enrichment effect”, with the CN groups concentrating at the resin―filler interface and effectively interacting with the surface functional groups on the BN particles, which resulted in an increase in the maximum bending stress. These findings emphasize the advantage of employing HSP methodologies in designing high-performance composite materials.

## 1 Introduction

In recent years, electronic components have been widely employed across various industries, including transportation, energy, and home appliances, increasing the demand for higher-performance and smaller components. Consequently, the heat generation density of electronic components continues to rise, making heat dissipation performance a critical determinant in their product lifetime and performance.^1,2^ In general, aluminum nitride and silicon nitride ceramics are utilized as heat sinks for automotive power devices^3−5^ because of their high thermal conductivity, electrical insulation, and better mechanical properties that enable them to withstand assembly stresses. Nonetheless, ceramic heat sinks present several challenges when bonded to a metal plate, typically copper, for efficient heat diffusion, including poor adhesion resulting in high thermal resistance at the interface between the heat sink and the metal plate, as well as high costs and complex processing requirements. By contrast, resin heat sinks^6,7^ exhibit better adhesion to copper, workability, and bending strengths compared to ceramic heat sinks; however, they have lower thermal conductivity. To address the issue, the concept of incorporating ceramic fillers into resins has been proposed.^2,8−11^

Boron nitride (BN) has garnered attention as a ceramic filler for superior heat dissipation due to its high thermal conductivity and electrical insulation properties.^12−14^ BN is typically supplied as a plate-like powder composed of covalently bonded nitrogen and boron in the c-plane, forming a structure with six-membered rings stacked in the *c*-axis direction and weakly bound together by van der Waals forces between the *c*-planes. This crystalline anisotropy results in a thermal conductivity of a few W/m K in the *c*-axis direction (perpendicular to the powder plane) and values as high as ∼200 W/m K in the *a*-axis direction (parallel to the powder plane) with covalent bonds.^15,16^

Therefore, the challenge lies in addressing the thermal conductivity anisotropy in order to effectively utilize BN as an active heat dissipation filler. One proposed solution is the implementation of a honeycomb-like structure.^17,18^ Heat-dissipating materials with a honeycomb-like structure are composite materials where platelike particles, such as BN, are arranged around a particulate resin, creating a continuous network of BN that exhibits high thermal conductivity. However, owing to the nonuniform structure of such composites, stress becomes concentrated at the interface between the resin and BN. Since the bending stress in these structured composites tends to be lower than that for nonstructured (uniform) composites with uniformly dispersed filler composites, structured composite heat sinks must overcome the trade-off between high thermal conductivity and sufficient bending strength to withstand the process of attachment to the module.

On the basis of this background, the present study proposes a composite material with a honeycomb-like structure that exhibits high thermal conductivity and improved mechanical properties achieved through material design by controlling the affinity between the BN particles and resin from a surface chemistry perspective. The material design was initiated using a Hansen solubility parameter (HSP) evaluation. An HSP is a three-component solubility parameter based on cohesive energy density (defined as δ^2^ = *E*/*V*_mol_, where δ is Hildebrand’s solubility parameter, *E* is the enthalpy of vaporization, and *V*_mol_ is the molar volume of the substance).^19^ It finds application in various fields, including paints, cosmetics, and foods, where the control of affinity between two substances during, for example, dissolution, swelling, and particle dispersion, is required.^19−29^ The three terms of HSP are δ_D_: dispersion term, δ_P_: polarity term, and δ_H_: hydrogen bonding term. The Hildebrand’s solubility parameter is related to HSP via δ = (δ_D_^2^ + δ_P_^2^ + δ_H_^2^)^1/2^. If the HSP value for each substance is known, the affinity between them can be calculated and predicted.

Composite materials with a honeycomb-like structure were fabricated using BN particles and poly(methyl methacrylate) (PMMA) particles, which are commonly used as a thermoplastic resin, and their thermal conductivity, electrical resistance, and maximum bending strength were systematically measured. The HSP evaluation of BN particles showed that the surfaces of the BN particles were covered with strongly polar (high-δ_P_) functional groups. Therefore, it was hypothesized that partially improving the polarity of PMMA (i.e., modifying a part of PMMA to match the surface polarity of BN particles) can enhance their affinity, targeting that one of the famous HSP-derived concepts, “like seeks like”, effectively works at their interface. To investigate this, highly polar nitrile (CN) groups^19^ were introduced into PMMA, and their effects on the affinity to BN and mechanical properties (maximum bending stress) were examined. Furthermore, the phenomena at the contact interface were investigated using the surface HSP evaluation method proposed by Murase and Nakamura.^30^

## 2 Experimental Section

Sections 2.1 and 2.2 present the HSP evaluation procedures for granular PMMA and BN, respectively. This led to the idea of introducing CN groups into PMMA(CN-PMMA). Section 2.3 illustrates the procedures for preparing granular PMMA and CN-PMMA. Subsequently, in Section 2.4, the process of creating composite materials by mixing the resin particles obtained from Section 2.3 with BN is outlined. Sections 2.5 and 2.6 detail the procedures for surface HSP evaluation. Section 2.5 demonstrates the resin membrane preparation procedure for evaluation, while Section 2.6 outlines the method for determining surface HSP through contact angle measurements.

### 2.1 HSP Evaluation of PMMA Resin by the Dissolution Experiment

Samples of the 43 types of organic solvent (probe liquids, 2.0 mL each) listed in Table S1 were mixed with 0.2 g of the synthesized PMMA resin (see next section for preparation) and allowed to stand. The solvents were visually inspected to determine whether the resin dissolved (Score = 1) or remained undissolved (Score = 0). A photograph of the solution after the dissolution experiment has been presented in Figure S1. The results obtained were analyzed using the Hansen sphere method implemented in the software HSPiP,^31^ enabling the determination of HSP (including δ_D_, δ_P_, δ_H_, as well as interaction radius, *R*_0_).

The suitability of the interactions between two substances, characterized by their respective HSPs as (δ_D1_, δ_P1_, δ_H1_) and (δ_D2_, δ_P2_, δ_H2_), can be determined using the HSP distance, *R*_a_, through eq 1.
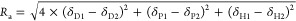
1

A larger (smaller) value of *R*_a_ indicates poor (good) compatibility. *R*_a_ can also be described as the delta between the HSP vectors in the 3D HSP space, i.e., Cartesian coordinate with the three axes of δ_D_, δ_P_, and δ_H_. In the analysis using the sphere method of polymer dissolution data, the probe liquids were classified as good solvents or poor solvents, and their HSP values were plotted in HSP space. In 3D HSP plots, the polymer HSP (HSP sphere center value and radius) is determined by performing an optimal fitting using HSP spheres with *R*_0_ as the radius. HSP plots of good (poor) solvents must lie within (outside) the sphere. In other words, solvents with a *R*_a_ less than or equal to *R*_0_ for the polymer are deemed suitable solvents that dissolve the polymer.

### 2.2 HSP Evaluation of BN Particles by the Dispersion Experiment

Samples of the 49 probe liquids (2.0 mL each) shown in Table S2 were mixed and dispersed with 0.1 g of BN particles (sheared PT110 manufactured by Momentive); then, the sample dispersions were left undisturbed for up to 12 days to allow sedimentation to occur. The sedimentation time, *t,* which represents the height of the supernatant liquid at 5 mm, was determined by visual observation. The measured *t* is corrected by the density, ρ_L_ (g/cm^3^), and viscosity, η (mPa s), of the solvent to calculate the relative sedimentation time, RST, using the equation RST = *t* × (ρ_S_ – ρ_L_)/η, where ρ_S_ is the density of the BN particles.^19^

For single HSP particles (surface-controlled particles with simplex surface ligands), the sphere fitting method (as described for polymer-HSP determination) is primarily adopted to obtain reliable HSPs for particles. In the case of multiple-HSP particles, however, the conventional sphere fitting method might not be suitable for determining the HSPs of mosaic-surface particles with complex surfaces consisting of 3, 4, or more surface ligands. To determine multiple HSPs for mosaic-surface powders, a new log-fit method is proposed. This method utilizes the real numeric RST data obtained from multiple surface ligands. A new HSP distance index for multiple HSP mosaic-surface particles, *R*_a_^Harmonic mean^, is introduced using the harmonic mean mixing concept, shown in eq 2:
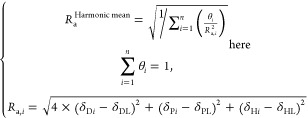
2where *n* is the number of surface ligands, (δ_D*i*_, δ_P*i*_, δ_H*i*_) are the HSP values for the *i*th surface ligand, (δ_DL_, δ_PL_, δ_HL_) are the HSP values for a probe liquid, θ_*i*_ is the surface coverage (or surface energy share) of the *i*th surface ligand, and *R*_a,*i*_ is the HSP distance from a probe liquid to the respective *i*th surface ligand. In other words, this harmonic mean mixing concept allows fitting multiple HSP spheres with variable interaction radii (θ_*i*_). In this study, assuming *n* = 4, the real numeric values of log (RST) were regressed linearly with respect to the *R*_a_^Harmonic mean^ to determine the multiple HSPs (δ_D*i*_, δ_P*i*_, δ_H*i*_) and their θ_*i*_ for the BN particles.

### 2.3 Preparation and Evaluation of Granular PMMA and CN-Incorporated PMMA (CN-PMMA)

#### 2.3.1 Materials

Poly(vinyl alcohol) (PVA, FUJIFILM Wako Pure Chemical Corporation, polymerization degree of 2000 and a saponification degree of 85%), methyl methacrylate (MMA, FUJIFILM Wako Pure Chemical Corporation), cyanoethyl acrylate (FUJIFILM Wako Pure Chemical Corporation), and azobis(isobutyronitrile) (AIBN, FUJIFILM Wako Pure Chemical Corporation) were used without purification.

#### 2.3.2 Preparation

Two grams of PVA were added to 210 mL of water with stirring. The mixture was heated to 90 °C and dissolved over a period of 1 h. After cooling to room temperature, the resulting solution was transferred to a round-bottom flask equipped with a mechanical stirrer. To the flask, 90 g of MMA and 0.9 g of AIBN were added. The contents of the flask were stirred at 300 rpm under nitrogen flow for 30 min. Polymerization was then carried out at 60 °C for 1 h, followed by an additional 10 h at 70 °C while maintaining the nitrogen flow and stirring. After cooling, the mixture was filtered through a mesh screen, washed with water, and subsequently washed with methanol. The resulting particles were then vacuum-dried and sieved through a 500 μm sieve, yielding 26 g of <500 μm PMMA particles.

The HSP evaluation of BN particles through dispersion experiments, as described above, revealed that the surface of BN particles had a high coverage of highly polar functional groups. Notably, one of these functional groups exhibited a close HSP similarity to CN groups,^31^ suggesting the potential to enhance the compatibility with BN. Consequently, CN groups were introduced into PMMA (CN-PMMA) in order to improve its affinity with BN.

**Figure 1:**
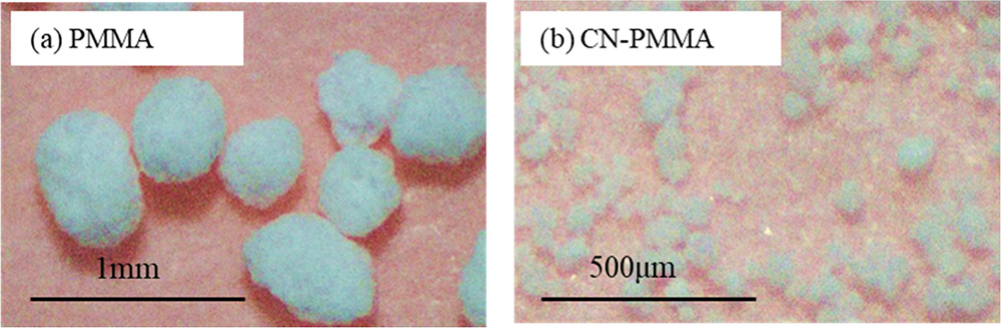
Photographs of fabricated (a) PMMA and (b) CN-PMMA resin particles.

MMA (76.5 g), 13.5 g of cyanoethyl acrylate, and 0.9 g of AIBN were added to the same aqueous PVA solution, and the temperature was raised to 70 °C while stirring in a similar manner. After polymerizing for 7 h with continuous stirring, the mixture was cooled to room temperature. Following the same procedure as during PMMA synthesis, the resulting mixture was subjected to filtration, washing, and drying. This yielded CN-PMMA particles containing 15 wt % of cyano monomer. The amounts of MMA and cyanoethyl acrylate were modified to obtain CN-PMMA particles with 10 and 5 wt % cyano monomer. The molecular structure of the prepared CN-PMMA is presented in Figure S1, and the appearance of the obtained resin is shown in Figure 1.

#### 2.3.3 Characterization

To determine the nitrogen content of each CN-PMMA sample, CHN elemental analysis was performed on the prepared PMMA particles with 0 to 15 wt % cyano monomer addition, enabling the calculation of the amount of CN groups (denoted by *n* in Figure S2). The PMMA particles and CN-PMMA particles were analyzed using the attenuated total reflection method using Fourier transform infrared spectroscopy (ATR-IR, Thermo Fisher Scientific). Additionally, PMMA and each CN-PMMA were swollen in chloroform, and the gel fraction was measured.

### 2.4 Preparation and Evaluation of Composite Materials Consisting of Resin and BN

#### 2.4.1 Materials

The same BN (sheared PT110 manufactured by Momentive) as used in Section 2.2 for HSP evaluation was employed along with the resin particles prepared in Section 2.3.

#### 2.4.2 Preparation

The BN particles and fabricated resin were weighed and mixed at a volume ratio of 1:1. Both PMMA and CN-PMMA were manually mixed with BN for 2 min. The mixture was then poured into a rectangular mold measuring 4 × 16 × 20 mm. The mold was heated at 130 °C for 30 min. Then, the product was subjected to a surface pressure of 300 MPa using a hand press for 1 min, and subsequently cooled to room temperature. BN particles were not subjected to dispersion processing.

#### 2.4.3 Characterization

The thermal diffusivity of each composite material was measured using the laser flash method with a thermal conductivity measuring device (NETZSCH, LFA447NANOFLASH). The measurements were conducted in a direction perpendicular to the direction of pressure during molding. The thermal conductivity was then calculated by multiplying the obtained thermal diffusivity by the calculated sample density and calculated specific heat. The sample density was calculated from the volume ratio of resin and BN. The specific heat values applied were the same for CN-PMMA as well as PMMA.

The electrical resistance was measured using an ultrahigh resistance meter (ADVANTEST, R8340A). Silver paste was applied to both sides of the sample to form electrodes, and a voltage of 1 V was applied during the measurement.

The bending strength was measured through three-point bending tests with a support interval of 10 mm and a test speed of 1 mm/min. The bending stress was calculated on the basis of the measured bending load at fracture. The fracture surfaces were analyzed by scanning electron microscopy (SEM) using Hitachi High-Technologies SU7000 and Regulus 8230 instruments. Energy dispersive X-ray spectroscopy (EDS) was performed using a Bruker QUANTAX FlatQUAD.

The composite materials were also characterized before fracturing through SEM (Hitachi High-Technologies SU3500). These SEM specimens were polished to reveal their cross-sectional surfaces, which were parallel to the direction of the thermal diffusivity measurement.

### 2.5 Preparation and Evaluation of Resin Film for Surface HSP Evaluation

In order to assess the improvement in affinity resulting from the incorporation of CN groups into PMMA, the surface HSP was measured using a technique based on contact angle measurements.^30^ This technique allowed the determination of the HSPs of the functional groups present on the surface, thereby elucidating the cause of the enhanced compatibility. To simulate the interaction between BN particles and CN groups, functional groups containing CN were attached to the surface of a Si substrate through silane coupling treatment. Subsequently, a resin film was prepared on the treated surface and then peeled off. The peeled-off surface of the resin film and the substrate surface were utilized for the HSP evaluation.

#### 2.5.1 Materials

A 20 × 20 mm Si substrates were utilized. Cyanoethyl trimethoxysilane (Gelest) was chosen as the silane coupling agent, and tetrahydrofuran (THF, FUJIFILM Wako Pure Chemical Corporation) was used for slurry adjustment. Other chemicals used were the same as in Section 2.3.

#### 2.5.2 Preparation

The silane-coupling surface treatment involved the following steps. After cleaning the Si substrate with acetone, the surface was UV-treated for 30 min and subsequently secured in a Teflon container. A vial was placed inside this Teflon container, and 25 μL of cyanoethyl trimethoxysilane was added to the vial under an Ar atmosphere. The container was sealed, and the vial was heat-treated in a muffle furnace at 150 °C for 30 min. The treated substrates were then ultrasonically cleaned in acetone to remove any unreacted materials, followed by drying on a hot plate at 50 °C to obtain CN-modified Si substrates (CN-Si substrates).

The following procedure was employed to prepare the solution for polymerizing the resin and forming the resin film. One gram of MMA, 0.01 g of AIBN, and THF were combined in a vial and stirred at 60 °C for 24 h to create a PMMA polymerization solution. Subsequently, a resin film was formed on the CN-Si substrate using spin coating. The PMMA polymerization solution was deposited onto the CN-Si substrate and rotated at 150 rpm for 3 min, followed by rotation at 1000 rpm for 5 min. The resulting product was then dried on a hot plate at 50 °C for at least 30 min.

CN-PMMA resin films were prepared using the identical procedure. For the CN-PMMA polymerization solution, 0.1 g of cyanoethyl acrylate was added to a vial containing 1 g of MMA, 0.01 g of AIBN, and 9 g of THF. The mixture was then stirred at 60 °C for 24 h, resulting in the formation of the CN-PMMA polymerization solution. The CN-PMMA polymerization solution was applied to the substrate and rotated at 150 rpm for 5 min, followed by rotation at 1000 rpm for 5 min. Finally, the film was dried on a hot plate at 50 °C for at least 30 min. The dried PMMA and CN-PMMA resin films were peeled off by using adhesive tape.

#### 2.5.3 Characterization

The X-ray photoelectron spectroscopy (XPS) analysis was conducted to determine the surface atomic composition of the Si substrate, CN-Si substrate, peeled-off PMMA film, and peeled-off CN-PMMA film. The measurements were performed using a Versaprobe II (ULVAC-PHI) instrument equipped with an Al Kα radiation source under a field-of-view diameter of 100 μm and a detection angle of 45°. To investigate the bonding of functional groups on the surface, the peeled-off resin film’s surface was analyzed using ATR-IR.

### 2.6 Surface HSP Evaluation by Contact Angle Measurement

Multiple probe liquids were applied to the peeled-off surface of the resin, and the contact angle (θ) with the surface was measured. From the obtained θ, the HSPs of the solid surfaces were determined using Young’s eq (eq 3) and Nakamura–Murase’s eqs (eqs 4–6)^30^:
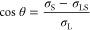
3
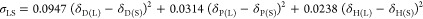
4
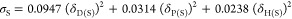
5
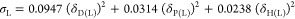
6where σ_S_ is the solid–air surface tension, σ_LS_ is the solid–liquid interfacial tension, and σ_L_ denotes the liquid–air surface tension. The HSP terms (dispersion term, polarity term, hydrogen bonding term) for the probe liquid are denoted by δ_D(L)_, δ_P(L)_, and δ_H(L)_, respectively. Table 1 lists the probe liquids applied to the peeled-off surface of each resin film along with their corresponding contact angles and HSPs. The surface HSP was determined by minimizing the deviations between the measured contact angles and the calculated ones based on eqs 3–6 while changing (optimizing) the surface HSP components (δ_D(S)_, δ_P(S)_, and δ_H(S)_) as fitting parameters.

**Table 1:** Probe Liquids and Their Contact Angles on the Peeled-Off Surfaces of PMMA and CN-PMMA Resin Films

probe liquid	HSP (MPa^1/2^)			contact angle (°)	
	δ_D_	δ_P_	δ_H_	PMMA	CN-PMMA
benzyl alcohol	18.4	6.3	13.7	**18.1**	**10.3**
1-bromonaphthalene	20.6	3.1	4.1	**19.8**	**30.8**
cis-decalin	17.6	0	0		**26.1**
*n*-decane	15.7	0	0	**0**	**16.9**
dimethyl sulfoxide	18.4	16.4	10.2		**12.2**
ethylene glycol	17	11	26	**58.7**	**60.0**
formamide	17.2	26.2	19	**65.3**	**63.5**
propylene carbonate	20	18	4.1		**9.7**
methyl benzoate	18.9	8.2	4.7		**8.1**
methylene iodide	22	3.9	5.5	**42.4**	**42.2**
nitromethane	15.8	18.8	6.1	**10.9**	**18.6**
tricresyl phosphate	19	12.3	4.5		**23.0**
water	15.5	16	42.3	**75.3**	**76.3**

## 3 Results and Discussion

### 3.1 HSP Evaluation of PMMA and BN by Dissolution and Dispersion Experiments

**Figure 2:**
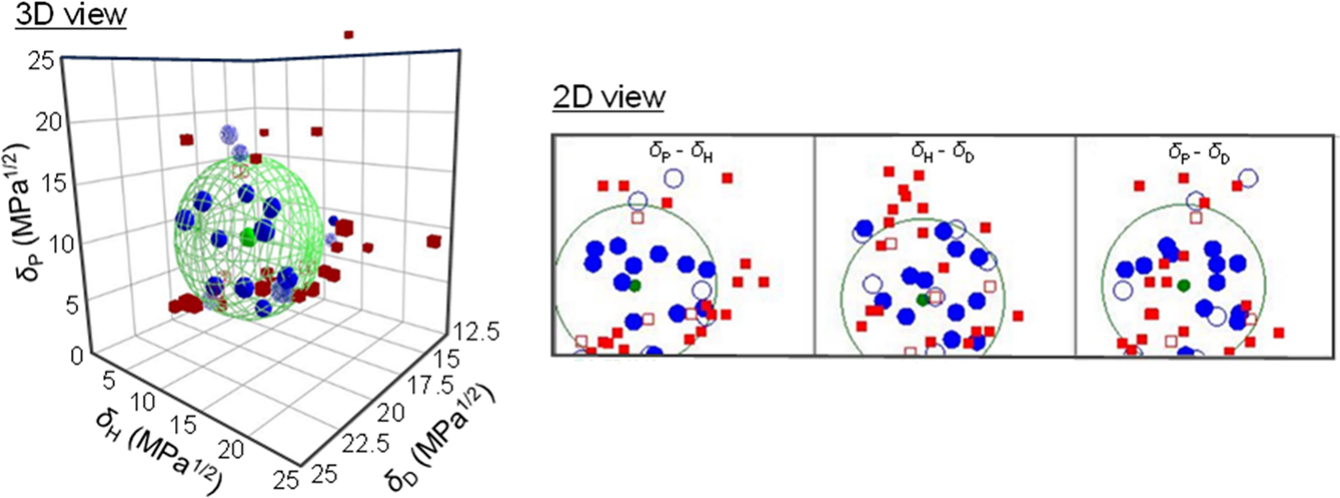
Quasi-3D plots of probe liquid and Hansen sphere of a PMMA in the HSP space (left), and 2D projection onto each plane (right). The HSP of the PMMA-dissolving (good) solvents (Score = 1, shown as blue solid circles inside the sphere and blue shaded (or blue open) circles outside the sphere), the HSP of the nondissolving (poor) solvents (Score = 0, indicated by red open squares inside the sphere and red solid squares outside the HSP sphere), and the obtained HSP and the interaction radius of PMMA (represented by a green solid circle and green wireframe sphere, respectively).

Figure 2 depicts the results of the Hansen sphere analysis for PMMA using the software HSPiP. The blue solid/blue shaded plots inside/outside the Hansen sphere (green wireframe sphere) represent good solvents that dissolved PMMA, while the red solid/red open plots outside/inside indicate poor solvents that did not dissolve PMMA. The center of the sphere represents the calculated HSP values. In the vicinity of the sphere’s shell, however, anomalous probe liquids were observed (indicated by red open squares inside the sphere and blue shaded circles outside the sphere, respectively). The fitting value (where 1 represents the best fitting quality without anomalies) fell acceptably good to 0.68, indicating that the obtained HSP would be quite reliable even though some anomalies arose. The HSPs obtained for PMMA from this Hansen sphere analysis were δ_D_ = 17.4 MPa^1/2^, δ_P_ = 8.5 MPa^1/2^, and δ_H_ = 7.1 MPa^1/2^, which are very consistent with previous reports on the HSPs of PMMAs.^19,31^

**Figure 3:**
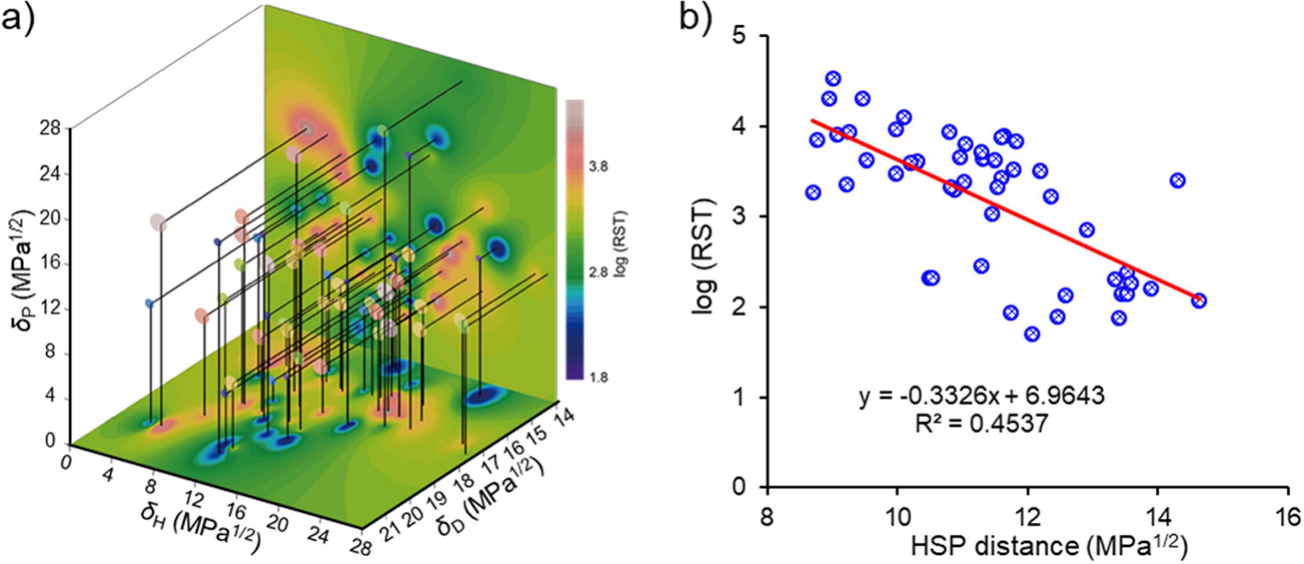
HSP evaluation results for BN particles. (a) Quasi-4D plots of probe liquids within the HSP space, (b) linear correlation between log(RST) values and *R*_a_^Harmonic mean^ in eq 2.

Figure 3 illustrates the results of the HSP analysis conducted on BN particles. Figure 3a displays quasi-4D plots of probe liquids in the HSP space, along with their projections onto the δ_P_ – δ_H_ and δ_D_ – δ_H_ planes. The log(RST) values for each solvent are color-coded. The good dispersion solvents in the HSP space are not concentrated in a single area but spread across multiple locations, making analysis via single-sphere HSP challenging.

To overcome this challenge, a linear regression analysis was performed utilizing eq 2 to establish the best correlation between *R*_a_^Harmonic mean^ and log(RST) values, assuming multiple (*n* = 4) surface-ligand HSPs. Figure 3b demonstrates the obtained linear correlation between log(RST) values with respect to the *R*_a_^Harmonic mean^. Table 2 lists the HSPs for multiple surface ligands and their corresponding surface coverage, achieved upon obtaining this optimal correlation.

**Table 2:** Correlated HSP Values and Surface Coverage of the Predicted Surface Functional Groups

possible surface ligands	HSP (MPa^1/2^)			coverage
	δ_D_	δ_P_	δ_H_	
nitrile/cyclic ester	17.6	19.2	2.4	29%
alcohol/diol	17.1	5.8	18.4	9%
carbonyl/amide	16.9	6.9	7.2	9%
urea/salt	18.9	20.1	26.9	52%

The HSPs for the surface ligands were utilized to distinguish each ligand, revealing that approximately 29% of BN particle surfaces consist of functional groups with HSPs comparable to those of nitrile or cyclic ester groups.^31^ Additionally, around 52% of the surface contains functional groups with HSPs resembling those of urea or ammonium salts.^31^ The validation of this multiple-HSP derivation method will be elaborated elsewhere.^32^ These results indicate that the surface of BN particles exhibits a high coverage of highly polar functional groups, suggesting that enhancing the polarity of the PMMA resin surface could improve their affinity. To validate this hypothesis, the effect of introducing highly polar CN groups into PMMA was investigated.

### 3.2 Material Characterization of Composites

**Figure 4:**
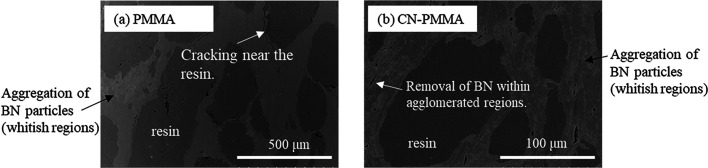
Cross-sectional SEM image of the honeycomb-like structured composite material consisting of BN particles (as a filler) and the matrix of (a) PMMA or (b) CN-PMMA resin. The dark areas represent resin particles, while the whitish regions indicate the agglomerated portions of the BN particles.

The cross-sectional SEM image of the composite material consisting of BN and PMMA is shown in Figure 4. A clear distinction is observed between the resin matrix and the aggregated BN particles, confirming the successful formation of the desired honeycomb-like structure.

Table 3 summarizes the results of material characterization. CHN elemental analysis revealed that the CN group contents (*n*) of PMMA and CN-PMMA were below the detection limit: 0.04, 0.06, and 0.11 for CN charging amounts of 0, 5, 10, and 15% by weight, respectively. *n* increased proportionally with the amount of CN charging, confirming the successful synthesis of PMMA modified with CN groups.

**Table 3:** Characterization Results for Fabricated Composite Material

parameters	amount of incorporated CN groups (wt %)			
	0	5	10	15
amount of CN groups in the resin, *n*	<0.02 (below detection limits)	0.04	0.06	0.11
thermal conductivity (W/m K)	36	34	30	26
electrical resistivity (Ω cm)	3.95 × 10^13^	9.64 × 10^13^	3.34 × 10^13^	1.00 × 10^14^
maximum bending stress (MPa)	3.7	5	7.1	5.2

**Figure 5:**
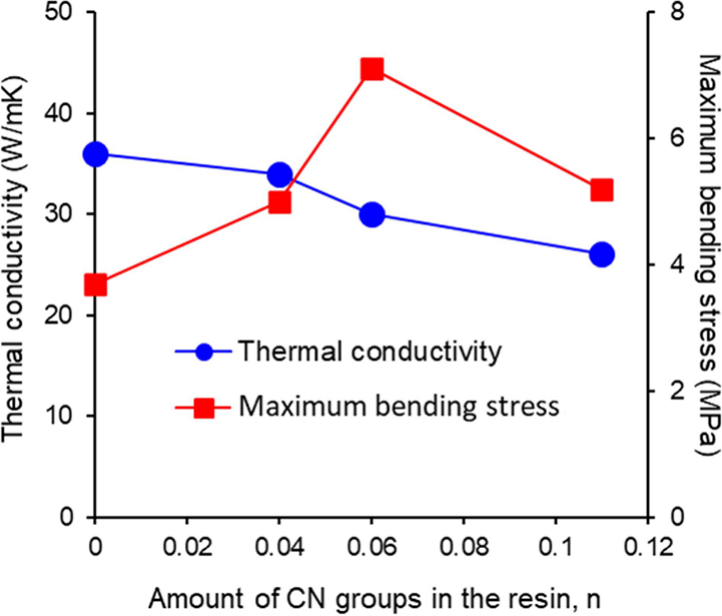
Maximum bending stress and thermal conductivity as a function of the amount of CN groups, *n*.

Figure 5 shows the maximum bending stress and thermal conductivity as a function of the amount of CN groups, *n*. The maximum bending stress was significantly improved in the samples with CN groups. The composite materials with added CN groups showed a maximum bending stress that was twice as high as that of the materials without CN addition. While differences in the resin particle size could be cited as a reason for the increase in maximum bending stress, no correlation was found between the resin particle size and maximum bending stress in this composite material (Table S3). This led us to conclude that control of interfacial functional groups is effective in increasing the maximum bending stress.

The maximum bending stress increased as *n* increased, reaching 7.1 MPa at *n* = 0.06 but showed a decreasing trend as *n* increased further. To investigate the cause of this trend, gel fraction measurements were conducted (Table S4). It was found that the gel fraction increased with increasing *n*. From these results, it can be inferred that as the amount of CN groups increases, the resin becomes more prone to cross-linking, leading to decreased flowability. Consequently, during the molding process, the resin may not flow adequately between the BN particles within the BN agglomerates, resulting in decreased adhesion between the BN particles. The results demonstrated that the introduction of CN groups into PMMA improves the compatibility between the BN particles and the resin, resulting in an increase in maximum bending stress, provided the value of *n* is below 0.11.

The thermal conductivity of the composite material without CN addition was as high as 36 W/m K, despite containing only 50 vol % BN particles. Wakashima’s equation^33^ predicts that achieving a thermal conductivity of 30 W/m K typically would require the addition of more than 80 vol % BN particles. Indeed, there have been reports of achieving a thermal conductivity of 30 W/m K with the addition of approximately 80 vol % BN.^34^ Such a high thermal conductivity was obtained owing to the formation of a honeycomb-like structure. When CN was added, the thermal conductivity decreased as the value of *n* increased. However, even at *n* = 0.11, the thermal conductivity remained at 28 W/m K without a sharp decline (Figure 5). One factor affecting the decrease in thermal conductivity with increasing *n* is the reduction in resin particle size (Figure 1). In this composite material, larger particles are known to exhibit higher thermal conductivity, as indicated in Table S3. It is believed that larger resin particles enhance the interparticle contact within BN agglomerates, thereby increasing the thermal conductivity. All samples exhibited an electrical resistivity of 1 × 10^13^ Ω cm or above. These results indicate that the composite materials fabricated with the addition of CN groups led to a noticeable improvement in maximum bending stress but had almost no drawback in terms of thermal conductivity or electrical resistivity.

**Figure 6:**
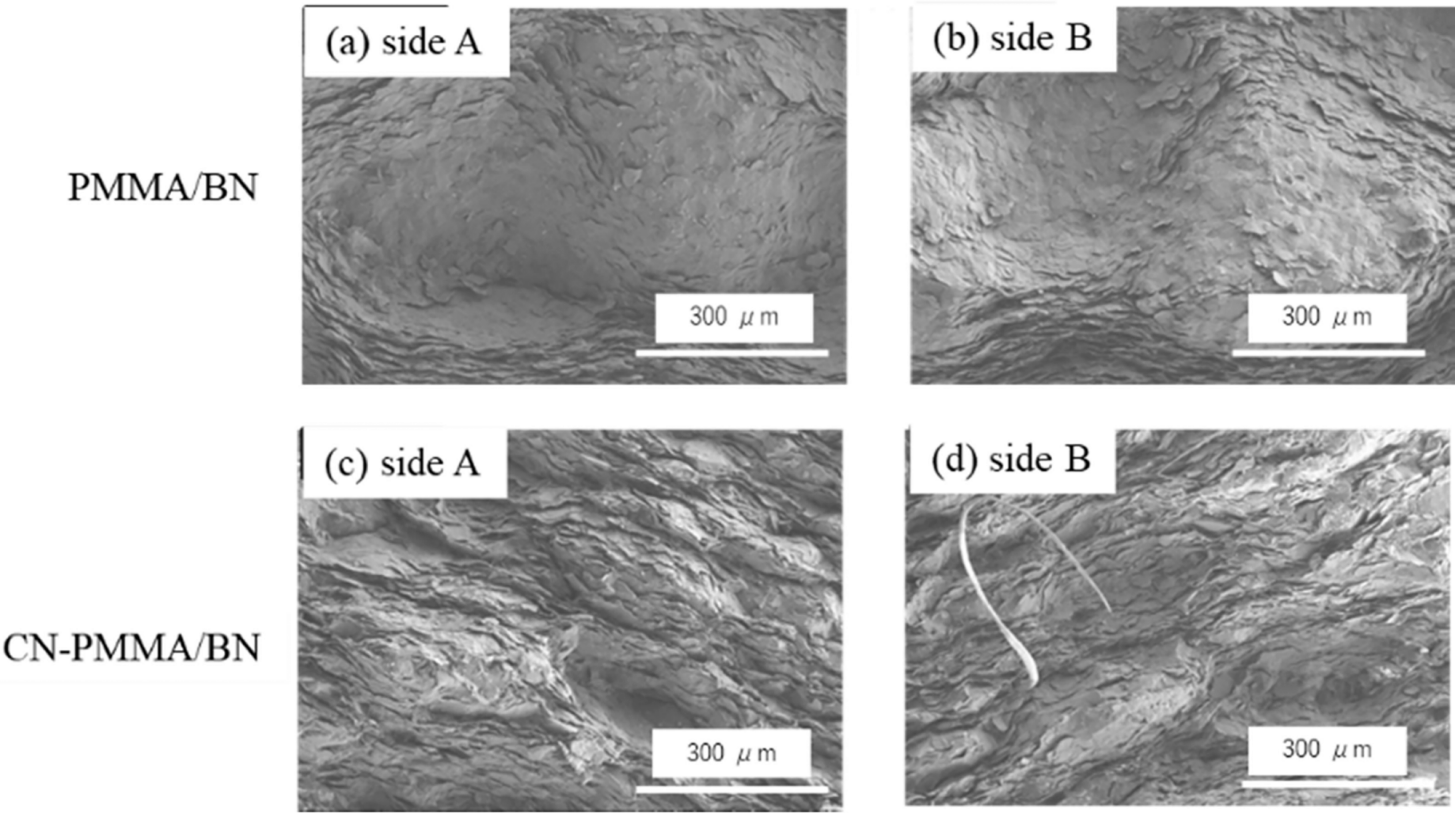
SEM fractographs of each composite material. The fracture surfaces on either side of the fractured region were observed, labeled “side A” and “side B”, respectively. (a) PMMA/BN, side A; (b) PMMA/BN, side B; (c) CN-PMMA/BN, side A; and (d) CN-PMMA/BN, side B.

**Figure 7:**
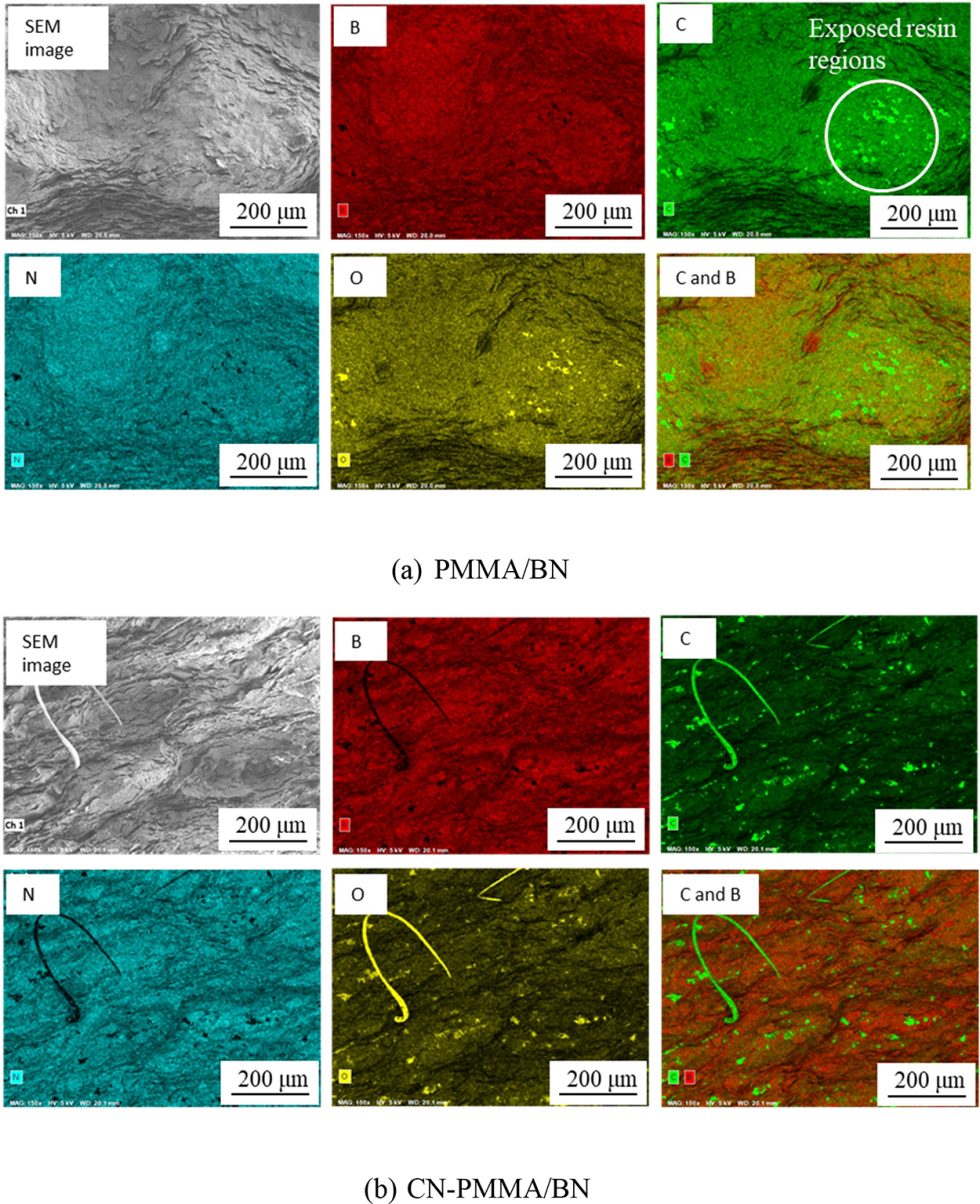
SEM fractographs (side B) of the fabricated composites and elemental mapping by EDS. (a) PMMA/BN and (b) CN-PMMA/BN.

**Figure 8:**
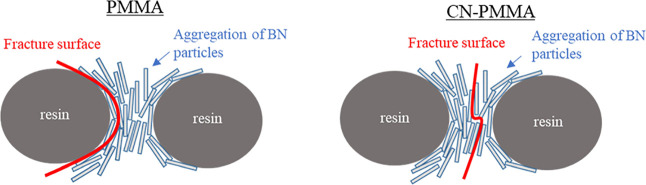
Orientation of fracture surface for each composite material.

Figure 6 is an SEM fractograph taken after the three-point bending test, while Figure 7 shows the results of EDS analysis on the area depicted in Figure 6. In the fracture surface of the composite material using PMMA, the *c*-plane of BN particles conforms to the shape of the PMMA particles (Figure 6a, b). Elemental analysis conducted by EDS revealed that most of the fracture surface was covered with BN. However, areas of exposed resin were observed near concave areas of the resin (Figure 7a). This suggested that fracture occurred near the resin, as illustrated in Figure 8 (left). A cross-sectional image taken before the fracture (see Figure 4a) also shows cracks near the resin, indicating that the fracture was initiated at these cracks. We suppose that the cracks formed at the interface between the BN and resin and then extended into the interfaces between BN and resin or the BN agglomerates near the resin.

On the other hand, in the composites with CN-PMMA (Figure 6c, d), the majority of BN particles are aligned, with their *c*-plane oriented perpendicular to the PMMA, which is quite different from the arrangement observed in the case of PMMA. The results of the elemental analysis conducted by EDS show that, similarly to the composite with PMMA, most of the area was covered with BN; however, there were no regions where the resin was concentrated and exposed (Figure 7b). Thus, we conclude that fracture occurred inside the agglomerate of BN particles (Figure 8, right), unlike in the case of PMMA. In the cross-sectional SEM image taken before fracture (see Figure 4b), a partial detachment of BN particles from the BN agglomerates can be observed (particles likely fell off during surface polishing), which were the weakest points in the composite material. Therefore, these points must have been the fracture initiation sites.

These results suggest that while the fracture is initiated at the interface between the resin and BN particles in composites with PMMA. When CN groups are introduced into PMMA, which improves the adhesion between the resin and BN particles, the fracture initiation site shifts to the BN particle agglomeration. Thus, SEM observation confirms that introducing CN groups into PMMA enhances the affinity between the resin and BN particles, leading to improved adhesion and maximum bending stress.

### 3.3 XPS and ATR-IR Measurement Results of Peeled-Off Resin Film

Table 4 lists the elemental compositions of the peeled-off surfaces of resins and the substrate surfaces, as calculated from XPS areal peak intensities with atomic sensitivity factors (see wide-scan XPS spectra in Figure S3). A significant amount of nitrogen (N) was detected on the CN-Si substrate, while no N was detected at all on the nontreated Si substrate. This confirmed that the CN groups were attached to the topmost surface of the CN-Si substrate through silane coupling treatment. N was also detected in the CN-PMMA film. The CN content based on the detected amount of N was approximately 10 wt %, which was equivalent to the actual amount of CN addition (9 wt %). In other words, conventional XPS measurement could not confirm that there was enrichment of CN groups on the resin surface (i.e., at the CN-Si/resin interface).

**Table 4:** Elemental Composition of Surface, as Calculated From XPS Spectra

samples	elemental composition (at%)				
	C	N	O	F	Si
Si substrate	6.99	0	40.44	0	52.57
CN-Si substrate	12.55	1.82	42.42	1.78	41.43
PMMA peeled-off film	76.74	0.2	22.34	0.05	0.68
CN-PMMA peeled-off film	77.83	1.36	20.58	0	0.24

In addition to N, fluorine (F) was detected on the CN-Si substrate and was presumably an impurity introduced from the silane coupling treatment environment. From the results for the peeled-off resin film, the atomic composition of Si was minimal, indicating that the transfer of Si from the substrate through peeling was negligible. The atomic composition of F was also negligible, confirming the absence of F contamination from the substrate.

**Figure 9:**
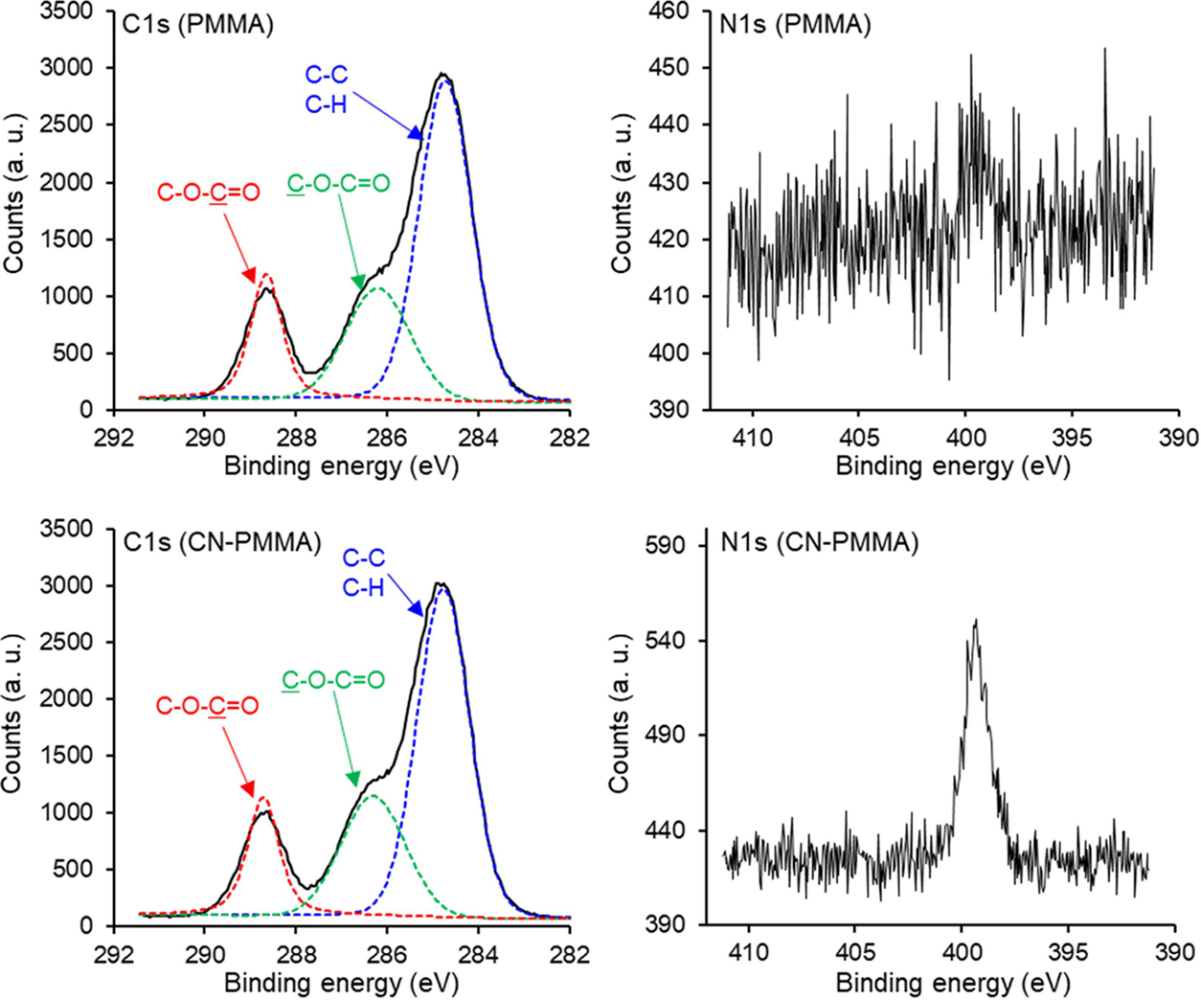
XPS spectra of C 1s and N 1s in the peeled-off PMMA resin film and CN-PMMA resin film. The black line represents the measured values, the red (around 288.7 eV, C–O–C=O) and green (around 286.3 eV, C–O–C=O) lines indicate the fitting values of peaks derived from C–O–C=O, while the blue (around 284.7 eV) line shows the fitting values of peaks attributed to C–C and C–H bonds.

The spectra of C 1s and N 1s near the PMMA peeled-off film and CN-PMMA peeled-off film are shown in Figure 9. In the C 1s spectrum, peaks due to C–C as well as C–H bonds around 284.7 eV, peaks attributed to the carbon, bonded to a single oxygen, of the **C**–O–C=O functional group around 286.3 eV,^35,36^ and peaks due to the carbon, bonded to two oxygens, of the C–O–**C**=O functional group around 288.7 eV^35,36^ were observed for both the peeled-off CN-PMMA resin film and PMMA resin film. In contrast, in the N 1s spectrum, a peak attributed to CN bonds, which was not observed in the PMMA peeled-off film, was detected at 399.3 eV^35,36^ in the peeled-off CN-PMMA film. These results suggest the presence of CN groups on the surface of the peeled-off CN-PMMA resin film, confirming that the desired material was obtained.

**Figure 10:**
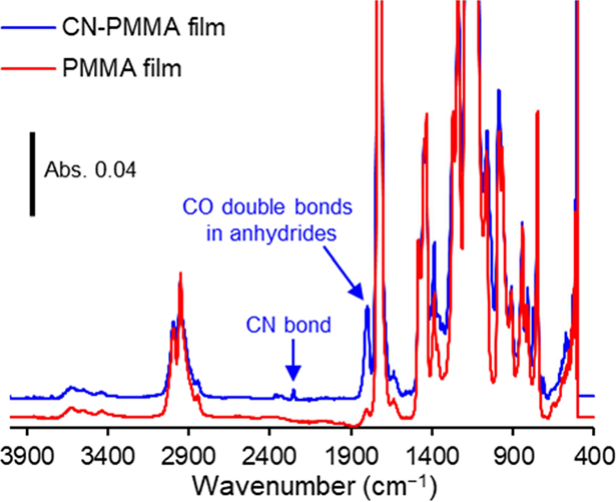
ATR-IR measurement results of the peeled-off resin films. The introduction of CN groups and the presence of anhydrides (impurities) were confirmed. Peaks other than those belonging to the CN groups and anhydrides were all from PMMA.

Figure 10 presents the ATR-IR measurement results for the peeled-off resin films. In the CN-PMMA film, a peak (around 2250 cm^–1^) attributed to CN bonds, which is not observed in the peeled-off PMMA film, was significantly detected, confirming the presence of CN groups, as also indicated by the XPS results. Additionally, peaks around 1800 and 1040 cm^–1^ were speculated to originate from, respectively, a C=O double bond and (CO)–O–(CO) bonds in anhydrides^37^ and were observed in the peeled-off CN-PMMA film. This functional group is different from the CN groups and functional groups derived from the PMMA matrix, suggesting the presence of secondary generated functional groups. Although these functional groups could not be identified exactly, they are likely from peroxides originating from the solvent THF.

**Figure 11:**
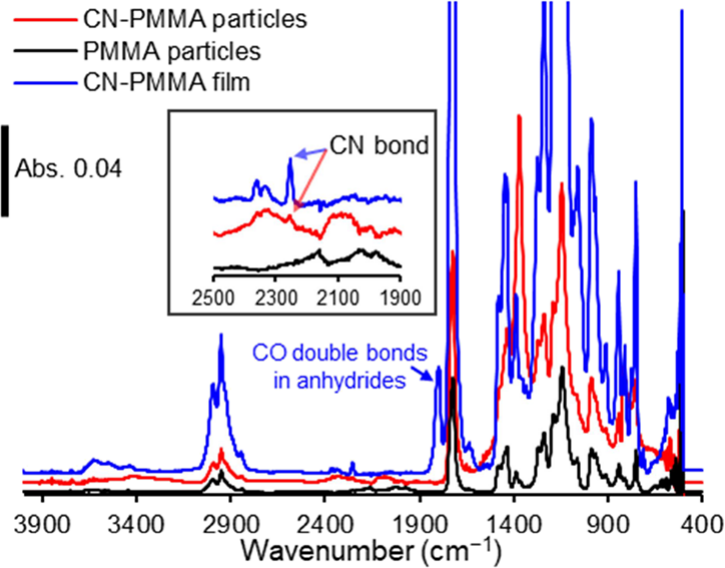
ATR-IR measurement results of the resin particles and CN-PMMA peeled-off resin film. The inset figure shows an enlarged view of the range between 1900 and 2500 cm^–1^ in wavenumbers.

Figure 11 shows the ATR-IR measurement results of the resin particles and CN-PMMA peeled-off resin film. No peaks due to anhydrides were observed in the resin particles, while the CN bond was confirmed in the CN-PMMA particles.^38^ These results clearly prove that impurities presumed to be anhydrides were not present in the composite material; therefore, not anhydrides but CN groups should contribute to the enhanced mechanical strength of the BN/CN-PMMA composites.

### 3.4 Evaluation of Surface HSP

The results of surface HSP measurement for the PMMA peeled-off film and CN-PMMA peeled-off film are listed in Table 1. The calculated HSPs for PMMA were as follows: δ_D(S)_ = 19.12 MPa^1/2^, δ_P(S)_ = 9.95 MPa^1/2^, and δ_H(S)_ = 5.58 MPa^1/2^. These are comparable to the results obtained when evaluating the HSP for PMMA by dissolving it in probe liquids (δ_D_ = 17.4 MPa^1/2^, δ_P_ = 8.5 MPa^1/2^, δ_H_ = 7.1 MPa^1/2^, as shown in Figure 2). Thus, these values have been validated for PMMA.

XPS as well as ATR-IR analysis revealed that the CN-PMMA peeled-off film assumed three functional groups: functional groups derived from the PMMA matrix, CN groups, and secondary generated functional groups. The HSPs derived from the PMMA matrix were held constant as specified above and used for the analysis. Consequently, the potential HSPs that may describe the surface of the CN-PMMA peeled-off film were as follows:

Ligand #1 (Functional groups derived from the PMMA matrix): δ_D(S)_ = 19.12 MPa^1/2^, δ_P(S)_ = 9.95 MPa^1/2^, δ_H(S)_ = 5.58 MPa^1/2^

Ligand #2: δ_D(S)_ = 15.51 MPa^1/2^, δ_P(S)_ = 16.81 MPa^1/2^, δ_H(S)_ = 10.05 MPa^1/2^

Ligand #3: δ_D(S)_ = 19.09 MPa^1/2^, δ_P(S)_ = 19.46 MPa^1/2^, δ_H(S)_ = 9.83 MPa^1/2^

The HSPs for Ligand #2 closely match those for acetonitrile (δ_D_ = 15.3 MPa^1/2^, δ_P_ = 18.0 MPa^1/2^, δ_H_ = 6.1 MPa^1/2^), indicating the presence of CN groups.^31^ Ligand #3 is thought to represent the HSPs for secondary generated functional groups. The selection of the functional groups among these that will cover the surface is determined by wetting adaption, whereby the surface structure and interfacial energy can be reversibly altered upon contact with a liquid.^39^

For instance, when using probe solvents (benzyl alcohol, 1-bromonaphthalene, dimethyl sulfoxide, propylene carbonate, methyl benzoate, methylene iodide, tricresyl phosphate) with smaller *R*_a_ values with respect to the PMMA matrix, the functional group of the PMMA matrix having smaller interfacial energies with respect to these solvents would cover the topmost surface, replacing another functional group that initially covered the topmost surface. Conversely, when using probe solvents (cis-decalin, *n*-decane, ethylene glycol, formamide, nitromethane, water) with larger *R*_a_ values (lower affinity) with respect to PMMA, the surface would remain enriched with CN groups. Thus, it can be inferred from these results that CN-PMMA deposited on a CN-Si substrate had an elevated concentration of CN groups on its surface.

However, the XPS results presented in the previous section show that the composition of CN groups was comparable to the feed composition, indicating the absence of significant surface enrichment. In the case of XPS, the analysis depth is a few nanometers; thus, XPS was unable to capture the surface enrichment at the topmost surface. This suggests that surface HSP evaluation, accompanied by contact angle measurement, should be much more sensitive to topmost surface functional groups than XPS, highlighting the advantage of HSP in this regard. Otherwise, the absence of significant surface enrichment might be due to the appearance of low-energy functional groups (hydrophobic parts) on the surface, as a result of the hydrophobic recovery effect.^40^

The functional groups on the resin surface emerge through wetting adaption, depending on their affinity with the adhering functional groups. The *R*_a_ between the surface functional groups of BN particles and the resin that may come into contact with the composite material was calculated (Table 5), thereby quantifying the affinity. As a result, when comparing the *R*_a_ for functional groups with HSPs resembling nitrile or cyclic ester groups and those with HSPs resembling urea or ammonium salts, where the BN surface coverage is high, *R*_a_ was smaller in CN groups than in functional groups of the PMMA matrix. From these results, we conclude that the introduction of CN groups into the resin, and thus enriching it at the resin/BN interface through the “like seeks like” effect in HSP terms or the “wetting adaption” effect in surface chemistry terms, improved the affinity with BN particles, which, in turn, enhanced adhesion as well as mechanical properties (maximum bending stress of the composites).

**Table 5:** *R*_a_ between Surface Functional Groups on BN and Surface Functional Groups on CN-PMMA^a^

component	*R*_a_ (MPa^1/2^)			
	possible surface functional groups of BN			
	nitrile/cyclic ester	alcohol/diol	carbonyl/amide	urea/salt
functional groups derived from the PMMA matrix	10.2	14.1	5.6	23.6
CN groups	9.0	14.2	10.7	18.5

a The *R*_a_ values were calculated from experimentally obtained HSPs via sedimentation time and contact angle measurements.

## 4 Conclusions

We successfully improved the adhesion and mechanical strength of composite materials used for heat sinks in automotive heat dissipation systems by elucidating/controlling the surface/interfacial functional groups using HSP evaluation. Surface functional groups of BN particles were identified using HSP evaluation, and to align these HSPs, CN groups were introduced into PMMA. The addition of CN groups increased the maximum bending stress for the samples, indicating improved adhesion between the resin and BN particles. SEM observations revealed that in the composite material using PMMA, fractures occurred near the interface between the resin and BN particles. In contrast, with the introduction of CN groups, the adhesion between the resin and BN particles improved, shifting the fracture initiation point to the agglomerates of BN particles. This suggests that the improved compatibility between the resin and BN particles due to the introduction of CN groups enhanced adhesion, leading to an increase in maximum bending stress. By incorporating a honeycomb-like structure, the composite material consisting of PMMA and BN particles exhibited a thermal conductivity of 36 W/m K. As the CN group content in CN-PMMA increased, the thermal conductivity of the composite material slightly decreased. However, even at a higher CN group content, the thermal conductivity remained at 28 W/m K without a significant drop. The presence of CN groups on the peeled-off CN-PMMA film was confirmed through XPS and ATR-IR measurements, which corresponded with the results of surface HSP evaluation. Furthermore, the surface HSP results indicated a higher concentration of CN groups on the surface, suggesting that topmost surface functional groups can be sensitively detected by contact angle measurements with surface HSP evaluation rather than XPS. HSP distance calculations demonstrated that the affinity between BN particles and CN-PMMA is significantly influenced by the presence of CN functional groups. In composite materials, it is believed that the affinity between CN functional groups and BN surface functional groups is higher when they come into contact, compared to the absence of CN functional groups. This is believed to contribute to an improvement in adhesive strength. The utilization of HSP offers promising prospects for advancements in material properties, including not only improvements in mechanical characteristics but also enhancements in adhesion and innovations in the material process design.

## Supporting Information Available

Detailed results of HSP evaluation for PMMA and BN; molecular formula of CN-PMMA; relationship between PMMA particle size and properties; result of gel fraction measurements; and wide-scan results (0–1100 eV) of XPS measurements (PDF)

## References

[ref1] Anandan S. S.; Ramalingam V. Thermal management of electronics: A review of literature. Thermal Science 2008, 12 (2), 5–26. 10.2298/TSCI0802005A.

[ref2] Huang X.; Jiang P.; Tanaka T. A review of dielectric polymer composites with high thermal conductivity. IEEE Electrical Insulation Magazine 2011, 27 (4), 8–16. 10.1109/MEI.2011.5954064.

[ref3] Mallik S.; Ekere N.; Best C.; Bhatti R. Investigation of Thermal Management Materials for Automotive Electronic Control Units. Applied Thermal Engineering 2011, 31, 355 10.1016/j.applthermaleng.2010.09.023.

[ref4] Yang B.; Peng C.; Song M.; Tang Y.; Wu Y.; Wu X.; Zheng H. Thermal Transport of AlN/Graphene/3C-SiC Typical Heterostructures with Different Crystallinities of Graphene. ACS Appl. Mater. Interfaces 2023, 15 (1), 2384–2395. 10.1021/acsami.2c17661. 36539985

[ref5] Duan W.; Li S.; Wang G.; Dou R.; Li W. G.; Zhang Y.; Li H.; Tan H. Thermal conductivities and mechanical properties of AlN ceramics fabricated by three dimensional printing. J. Eur. Ceram. Soc. 2020, 40 (10), 3535–3540. 10.1016/j.jeurceramsoc.2020.04.004.

[ref6] Prasher R. Thermal interface materials: Historical perspective, status, and future directions. Proceedings of the IEEE 2006, 94 (8), 1571–1586. 10.1109/JPROC.2006.879796.

[ref7] Kang D. G.; Park M.; Kim D. Y.; Goh M.; Kim N.; Jeong K. U. Heat Transfer Organic Materials: Robust Polymer Films with the Outstanding Thermal Conductivity Fabricated by the Photopolymerization of Uniaxially Oriented Reactive Discogens. ACS Appl. Mater. Interfaces 2016, 8 (44), 30492–30501. 10.1021/acsami.6b10256. 27762538

[ref8] Chen H.; Ginzburg V. V.; Yang J.; Yang Y.; Liu W.; Huang Y.; Du L.; Bin C. Thermal conductivity of polymer-based composites: Fundamentals and applications. Prog. Polym. Sci. 2016, 59, 41–85. 10.1016/j.progpolymsci.2016.03.001.

[ref9] Tsekmes I. A.; Kochetov R.; Morshuis P. H. F.; Smit J. J. Thermal conductivity of polymeric composites: A review. Proceedings of IEEE International Conference on Solid Dielectrics, ICSD; IEEE: Bologna, Italy, 2013, 678 681

[ref10] Pan X.; Debije M. G.; Schenning A. P. H. J.; Bastiaansen C. W. M. Enhanced Thermal Conductivity in Oriented Polyvinyl Alcohol/Graphene Oxide Composites. ACS Appl. Mater. Interfaces 2021, 13 (24), 28864–28869. 10.1021/acsami.1c06415. 34102056 PMC8289248

[ref11] Yang S.; Wang Q.; Wen B. Highly Thermally Conductive and Superior Electrical Insulation Polymer Composites via in Situ Thermal Expansion of Expanded Graphite and in Situ Oxidation of Aluminum Nanoflakes. ACS Appl. Mater. Interfaces 2021, 13 (1), 1511–1523. 10.1021/acsami.0c18603. 33347278

[ref12] Hutchinson J. M.; Moradi S. Thermal conductivity and cure kinetics of epoxy-boron nitride composites-A review. Materials 2020, 13 (16), 3634 10.3390/ma13163634. 32824496 PMC7476057

[ref13] Mazumder M. R. H.; Mathews L. D.; Mateti S.; Salim N. V.; Parameswaranpillai J.; Govindaraj P.; Hameed N. Boron nitride based polymer nanocomposites for heat dissipation and thermal management applications. Applied Materials Today 2022, 29, 101672 10.1016/j.apmt.2022.101672.

[ref14] Hu J.; Huang Y.; Yao Y.; Pan G.; Sun J.; Zeng X.; Sun R.; Xu J. Bin; Song B.; Wong C. P. Polymer Composite with Improved Thermal Conductivity by Constructing a Hierarchically Ordered Three-Dimensional Interconnected Network of BN. ACS Appl. Mater. Interfaces 2017, 9 (15), 13544–13553. 10.1021/acsami.7b02410. 28362080

[ref15] Duclaux L.; Nysten B.; Issi J.-P.; Moore A. Structure and low-temperature thermal conductivity of pyrolytic boron nitride. PHYSICAL REVIEW B 1992, 46, 3362–3367. 10.1103/PhysRevB.46.3362. 10004050

[ref16] Tanimoto M.; Yamagata T.; Miyata K.; Ando S. Anisotropic thermal diffusivity of hexagonal boron nitride-filled polyimide films: Effects of filler particle size, aggregation, orientation, and polymer chain rigidity. ACS Appl. Mater. Interfaces 2013, 5 (10), 4374–4382. 10.1021/am400615z. 23607623

[ref17] Agari Y.; Hirano H.; Kadota J.; Hasegawa K. Thermal Conductivity of Boron Nitride/Phenol Resin Composite with Honeycomb-like Structure. J. Netw. Polym. 2011, 32 (1), 10–18.

[ref18] Wu X.; Liu W.; Shi F.-G.; Yang L.; Zhang C. Constructing three-dimensional boron nitride network for highly thermally conductive epoxy resin composites. Polym. Compos. 2022, 43 (3), 1711–1717. 10.1002/pc.26490.

[ref19] Hansen C. M. Hansen Solubility Parameters: A User’s Handbook, Second Ed.; CRC Press: Boca Raton, 2007.

[ref20] Abbott S. Solubility, similarity, and compatibility: A general-purpose theory for the formulator. Curr. Opin. Colloid Interface Sci. 2020, 48, 65–76. 10.1016/j.cocis.2020.03.007.

[ref21] Fardi T.; Stefanis E.; Panayiotou C.; Abbott S.; van Loon S. Artwork conservation materials and Hansen solubility parameters: A novel methodology towards critical solvent selection. Journal of Cultural Heritage 2014, 15 (6), 583–594. 10.1016/j.culher.2013.11.006.

[ref22] Süß S.; Sobisch T.; Peukert W.; Lerche D.; Segets D. Determination of Hansen parameters for particles: A standardized routine based on analytical centrifugation. Advanced Powder Technology 2018, 29 (7), 1550–1561. 10.1016/j.apt.2018.03.018.

[ref23] Takai-Yamashita C.; Shinkai I.; Fuji M.; El Salmawy M. S. Effect of water soluble polymers on formation of Na_2_SO_4_ contained SiO_2_ microcapsules by W/O emulsion for latent heat storage. Advanced Powder Technology 2016, 27 (5), 2032–2038. 10.1016/j.apt.2016.07.012.

[ref24] Nakamura D.; Shigetoh K.; Suzumura A. Tantalum carbide coating via wet powder process: From slurry design to practical process tests. Journal of the European Ceramic Society 2017, 37 (4), 1175–1185. 10.1016/j.jeurceramsoc.2016.10.029.

[ref25] Nakamura D.; Hirano M.; Ohta R. Nontoxic organic solvents identified using an a priori approach with Hansen solubility parameters. Chem. Commun. 2017, 53 (29), 4096–4099. 10.1039/C7CC01434A. 28345101

[ref26] Tsutsumi S.; Kato Y.; Namba K.; Yamamoto H. Functional composite material design using Hansen solubility parameters. Results in Materials 2019, 4, 100046 10.1016/j.rinma.2019.100046.

[ref27] Murase M.; Riichiro O. Prediction of Molecular Affinity on Solid Surfaces via Three-Dimensional Solubility Parameters Using Interfacial Free Energy as Interaction Threshold. J. Phys. Chem. C 2019, 123 (21), 13246–13252. 10.1021/acs.jpcc.9b00154.

[ref28] Ma J.; Larsen R. M. Comparative study on dispersion and interfacial properties of single walled carbon nanotube/polymer composites using hansen solubility parameters. ACS Appl. Mater. Interfaces 2013, 5 (4), 1287–1293. 10.1021/am302407z. 23363469

[ref29] Raynal M.; Bouteiller L. Organogel formation rationalized by Hansen solubility parameters. Chem. Commun. 2011, 47 (29), 8271–8273. 10.1039/c1cc13244j. 21709882

[ref30] Murase M.; Nakamura D. Hansen Solubility Parameters for Directly Dealing with Surface and Interfacial Phenomena. Langmuir 2023, 39 (30), 10475–10484. 10.1021/acs.langmuir.3c00913. 37463335

[ref31] Abbott S.; Hansen C. M.; Yamamoto H. Hansen solubility parameters in practice, complete with eBook, software and data 5th ed., 2015, http://www.hansen-solubility.com (accessed 15 Dec. 2023.).

[ref32] Submitted to Langmuir, Manuscript ID: la-2024–006417, under review.

[ref33] Wakashima H.; Tsukamoto H. Mean-field micromechanics model and its application to the analysis of thermomechanical behavior of composite materials. Materials Science and Engineering: A 1991, 146 (1–2), 291–316. 10.1016/0921-5093(91)90284-T.

[ref34] Ishida H.; Rimdusit S. Very high thermal conductivity obtained by boron nitride-filled polybenzoxazine. Thermochim. Acta 1998, 320 (1–2), 177–186. 10.1016/S0040-6031(98)00463-8.

[ref35] Taylor A. Practical surface analysis, 2nd edn., vol I, Auger and X-ray photoelectron spectroscopy. Edited by D. Briggs & M. P. Seah, John Wiley, New York, 1990, 657 pp., price: £86.50. ISBN 0471 92081 9. J. Chem. Technol. Biotechnol. 1992, 53, 215 10.1002/jctb.280530219.

[ref36] Brucker C. F. Electron spectroscopy: Theory, techniques and applications 1982, 4 (6), i–ii. 10.1002/sia.740040611.

[ref37] Bellamy L. J. The Infra-red Spectra of Complex Molecules; Springer Dordrecht, 1975.

[ref38] Tarducci C.; Schofield W. C. E.; Badyal J. P. S.; Brewer S. A.; Willis C. Cyano-Functionalized Solid Surfaces. Chem. Mater. 2001, 13, 1800–1803. 10.1021/cm000810.

[ref39] Butt H. J.; Berger R.; Steffen W.; Vollmer D.; Weber S. A. L. Adaptive Wetting - Adaptation in Wetting. Langmuir 2018, 34 (38), 11292–11304. 10.1021/acs.langmuir.8b01783. 30110544

[ref40] Tadmor R. Open Problems in Wetting Phenomena: Pinning Retention Forces. Langmuir 2021, 37 (21), 6357–6372. 10.1021/acs.langmuir.0c02768. 34008988

