# Supplementary material for: Enhancement of Mechanical Properties of High-Thermal-Conductivity Composites Comprising Boron Nitride and Poly(methyl methacrylate) Resin through Material Design Utilizing Hansen Solubility Parameters
Source: ACS Appl Mater Interfaces. 2024 May 9;16(20):26653–63. doi: 10.1021/acsami.4c00626 (PMC11129141; doi:10.1021/acsami.4c00626)
Supplement: Supplementary file 1 — am4c00626_si_001.pdf [file am4c00626_si_001.pdf]

Supporting Information

Enhancement of Mechanical Properties of High-Thermal-Conductivity Composites comprising Boron Nitride and Polymethyl Methacrylate Resin through Material Design utilizing Hansen Solubility Parameters

Yumi Inagaki*, Masakazu Murase, Hiromitsu Tanaka, Daisuke Nakamura

Toyota Central R&D Laboratories, Inc., Nagakute, Aichi 480-1192, Japan
*Email: y-inagaki@mosk.tytlabs.co.jp

Table S1. Solvents used for PMMA dissolution experiment and results.

	Probe liquid	Score
N#1	Acetone	1
N#2	1-Butanol	0
N#3	Toluene	1
N#4	Ethanol	0
N#5	Propylene Carbonate	0
N#6	Glycerol	0
N#7	1,4-Dioxane	1
N#8	Dipropylene Glycol	0
N#9	Acetonitrile	0
N#10	Formamide	0
N#11	N-Methyl Formamide	0
N#12	Formic Acid	1
N#13	Methylene Diiodide (Diiodomethane)	0
N#14	Diethyl Ether	0
N#15	Cyclohexane	0
N#16	Nitrobenzene	1
N#17	Acetic Acid	1
N#18	1-Bromonaphthalene	0
N#19	γ-Butyrolactone (GBL)	1
N#20	Dimethyl Sulfoxide (DMSO)	0
N#21	Methylal(Dimethoxymethane)	1
N#22	Tetrahydronaphthalene	0
N#23	Pyridine	1

N#24	Dimethyl Acetamide (DMA)	1
N#25	Methyl Isobutyl Carbinol(4-Methyl-2-pentanol)	0
N#26	Xylene	0
N#27	Benzonitrile	1
N#28	1-Nitropropane	1
N#29	Caprolactone (Epsilon) 6-Hexanolactone	0
N#30	Thiazole	1
N#31	Benzothiazole	0
N#32	Aniline	1
N#33	Phenylhydrazine	0
N#34	Diethyl Amine	0
N#35	Benzyl Alcohol	1
N#36	Cyclohexanol	0
N#37	1-Hexanol 4-5	0
N#38	Salicylaldehyde	1
N#39	Bromoform	1
N#40	2-Chlorophenol(o-Chlorophenol)	1
N#41	Ethylenediamine	0
N#42	2-Butanol	0
N#43	Diethyl Sulfide(Ethyl Sulfide)	0

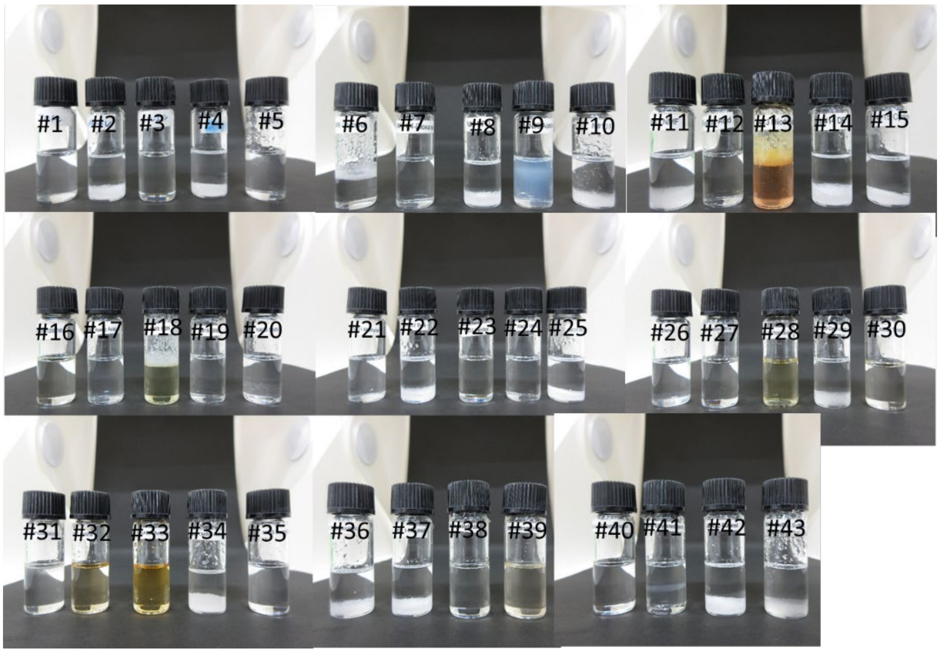

Figure S1. Photograph of the solution after the PMMA dissolution experiment.

Table S2. Solvents used for BN particle dispersion experiment and results.

	Probe liquid	t(s)	ρL (g/cm^3)	Visc. (mPa·s)	*RST* (arb. unit)
N#1	Dimethyl Carbonate	110	1.069	0.584	213
N#2	Diethylene Glycol Monobutyl Ether	12060	0.952	5.052	2979
N#3	Diethylene Glycol Monoethyl Ether	8700	0.984	3.828	2764
N#4	Acetone	1890	0.79	0.312	8541
N#5	Diethylene Glycol Monomethyl Ether	13320	1.017	3.565	4420
N#6	Ethylene Glycol Monoethyl Ether	410	0.93	1.838	283
N#7	Methyl Ethyl Ketone (MEK)	5460	0.805	0.378	20150
N#8	Ethylene Glycol Monomethyl Ether	2475	0.96	1.541	1992
N#9	n-Butyl Acetate	1238	0.87	0.678	2429
N#10	1-Butanol	4380	0.81	2.619	2325
N#11	Propylene Carbonate	39624	1.188	2.5	16040
N#12	2-Propanol	26030.4	0.781	2.015	18331
N#13	1,3-Dioxolane	3660	1.065	0.589	7053
N#14	1-Nitropropane	140	0.998	0.798	211
N#15	Glycerol	1099200	1.257	964	1075
N#16	Dimethyl Acetamide (DMA)	3380	0.937	0.945	4517
N#17	1,4-Dioxane	210	1.04	1.196	204
N#18	Cyclohexanone	3010.7	0.947	2.02	1868
N#19	Caprolactone (Epsilon)	61200	1.067	5.532	12534
N#20	Dipropylene Glycol	429480	1.0206	79.06	6407
N#21	Ethanol	3220	0.82	1.082	4107
N#22	Acetonitrile	2250	0.786	0.342	9303
N#23	Dimethyl Sulfoxide (DMSO)	160	1.1	1.991	88
N#24	2-Chlorophenol	275	1.241	3.376	78
N#25	Formamide	2230	1.13	3.322	718
N#26	N-Methyl Formamide	2310	1.011	1.65	1665
N#27	γ-Butyrolactone (GBL)	14280	1.125	1.745	8797
N#28	N-Methyl-2-Pyrrolidone (NMP)	3120	1.025	1.695	2163
N#29	Dimethyl Formamide	2060	0.944	0.8006	3232
N#30	Methanol	30	0.791	0.55	77
N#31	Ethylene Glycol	51216.7	1.113	16.3876	3397
N#32	1,3-Butanediol	345900	1.005	97.262	4250

N#33	Diethylene Glycol	194916	1.114	27.15	7797
N#34	Cyclohexanol	346080	0.963	56.5	7577
N#35	Benzyl Alcohol	9960	1.045	5.376	2140
N#36	Acetic Anhydride	5220	1.082	0.842	6931
N#37	Thiazole	140	1.2	1.007	139
N#38	Pyridine	5220	0.978	1.227	5199
N#39	Nitrobenzene	410	1.196	1.686	244
N#40	Water	120	0.998	0.89	162
N#41	Acetic Acid	135	1.049	1.115	139
N#42	Aniline	7920	1.022	3.69	2528
N#43	Formic Acid	280	1.214	1.51	183
N#44	Salicylaldehyde	320	1.146	2.501	135
N#45	Dichloroacetic Acid	32798.65	1.553	5.06	4194
N#46	Acetol	190	1.042	4.395	50
N#47	Ethanolamine	64740	1.014	19.342	3970
N#48	Ethylenediamine	6660	0.893	1.082	8045
N#49	Thiophenol	120	1.073	1.144	118

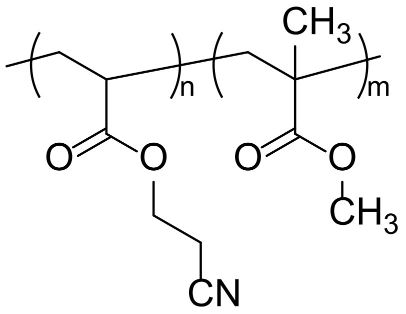

Figure S2. Molecular structure of PMMA with introduced CN groups.

Table S3. Maximum bending stress and thermal conductivity of composite materials for different particle sizes of PMMA.

Particle sizes of PMMA (μm)	36-150	255-355	More than 500
Maximum bending stress (MPa)	4.3	3.0	3.7
Thermal conductivity (W/mK)	27	27	36

Table S4. Gel fraction measurement results.

Amount of incorporated CN groups (%)	Amount of CN groups in resin *n*	Gel Fraction (%)
0	<0.02	0
5	0.04	17.4
10	0.06	53.1
15	0.11	80.0

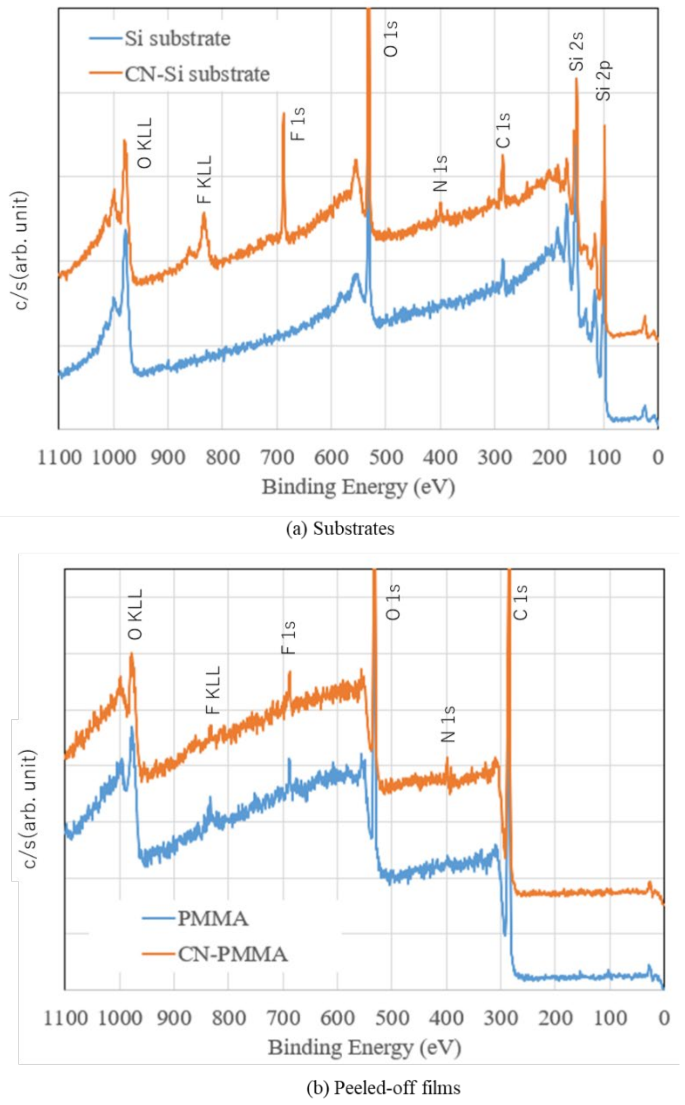

Figure S3. Wide-scan XPS measurement results for (a) substrates and (b) release resin films.
